# Modeling linkage disequilibrium in exact linkage computations: a comparison of first-order Markov approaches and the clustered-markers approach

**DOI:** 10.1186/1753-6561-1-s1-s159

**Published:** 2007-12-18

**Authors:** Cornelis A Albers, Hilbert J Kappen

**Affiliations:** 1 Department of Biophysics, Radboud University, 126 Geert Grooteplein 21, Nijmegen, Gelderland 6525EZ The Netherlands

## Abstract

Recent studies have shown that linkage disequilibrium (LD) between single-nucleotide polymorphism (SNP) markers is widespread. Assuming linkage equilibrium has been shown to cause false positives in linkage studies where parental genotypes are not available. Therefore, linkage analysis methods that can deal with LD are required to accurately analyze SNP marker data sets. We compared three approaches to deal with LD between markers: 1) The clustered-markers approach implemented in the computer program MERLIN; 2) The standard hidden Markov model (HMM) multipoint model augmented with a first-order Markov model for the allele frequencies of the founders, in which we considered both a Bayesian and a maximum-likelihood implementation of this approach; 3) The 'independent' SNPs approach, i.e., removing SNPs from the data set until the remaining SNPs have low levels of LD.

We evaluated these approaches on the Illumina 6K SNP data set of affected sib-pairs of Problem 2. We found that the first-order Markov model was able to account for most of the strong LD in this data set. The difference between the Bayesian and maximum- likelihood implementation was small. An advantage of the first-order Markov model is that it does not require the user to specify parameters.

## Background

In this paper we evaluate a number of approaches that explicitly model linkage disequilibrium (LD) between markers. These approaches are extensions of pedigree likelihood models such as the HMM described by Kruglyak et al. [1], which assumes linkage equilibrium between the markers. The goal is to analyze affected sib-pair families in order to localize genetic susceptibility loci of complex diseases.

## Methods

### Maximum likelihood with clustered markers

Abecasis et al. [2] proposed a clustered markers approach to model LD, which they implemented in the computer program MERLIN. The main idea is to cluster tightly linked markers and to model LD between the markers in a cluster by estimating haplotype frequencies with the expectation-maximization (EM) algorithm. The haplotype frequencies are estimated for each cluster independently. Markers are clustered together when the level of LD exceeds a threshold to be specified by the user. The approach requires that there are no recombination events between markers in a cluster. When the genotypes indicate an obligate recombinant between markers in a cluster, genotypes are set to missing to remove the obligate recombinant. Furthermore, the approach does not model LD *between *clusters.

### Independent markers approach

First, pair-wise LD correlation coefficients are estimated between pairs of markers. When the level of LD between a pair of markers exceeds a threshold, which must be specified by the user, one of the markers is removed from the data set. As a result, the remaining set of markers will not have markers with levels of LD that exceed the threshold.

### A first-order Markov model

We propose to use a first-order Markov model to handle LD between the markers; LD between each pair of markers is modeled with an approach similar to the one proposed by Yang et al. [3] for a marker and a trait. In contrast with their work, we will compute identity-by-descent (IBD) statistics such as *Z*_*pairs *_[1], and will not model LD between marker and trait alleles.

The first-order Markov model is applied to the alleles of each founder individual independently:

*P*(***G***_*i*_|*α*, *β*) = *P*(*G*^*l*^_*i*_|*α*^*l*^)∏_*l*=2,...,*L*_*P*(*G*^*l*+1^_*i*_|*G*^*l*^_*i*_, *α*^(*l*,*l*+1)^, *β*^(*l*,*l*+1)^),

where *i *is founder, where *G*^*l*^_*i *_represents an allele (which can be paternal or maternal) for marker *l *of a founder *i*, and *L *is the number of markers. *α *and *β *are the LD parameters that quantify the linkage between the alleles of two adjacent SNPs:

*P*(*A*|*a*, *α*^(*l*,*l*+1)^, *β*^(*l*,*l*+1)^) = *α*^(*l*,*l*+1)^, *P*(*A*|*b*, *α*^(*l*,*l*+1)^, *β*^(*l*,*l*+1)^) = *β*^(*l*,*l*+1)^.

In this equation, the alleles of marker *l *are denoted by (*A*, *B*); the alleles of marker *l *+ 1 are denoted by (*a*, *b*). Our approach assumes Hardy-Weinberg equilibrium between founder alleles. MERLIN and the independent markers approach also make this assumption. The goal is to infer the posterior probability distributions of segregation indicators of the non-founders (NF) for locus *l*, denoted by **s**_*NF *_^*l*^, given marker data, integrated over the nuisance parameters *α*^(*l*,*l*+1) ^and *β*^(*l*,*l*+1)^:

$$P({\text{s}}_{NF}^{l}|\text{M})=\int d\alpha \int d\beta P(\alpha ,\beta )\sum_{\text{G}}\sum_{{\text{s}}_{NF}\backslash {\text{s}}_{NF}^{l}}P({\text{s}}_{NF}|\text{G})P(\text{G}|\text{M})\prod_{i\in \{F\}}P({\text{G}}_{i}|\alpha ,\beta )$$

Here, *G*_*i *_with *iε*{*F*} represents the vector of paternal or maternal founder alleles for all marker loci; when multiple families are available, the product is implied to run over the founder alleles in all families. *P*(*G*_*i*_|*α*, *β*) is the first-order Markov model given by Eq. (1). Thus, the segregation indicators of individuals in different families become dependent through the jointly shared, unobserved model parameters ***α ***and ***β ***given marker data ***M***.

Evaluation of the integral in Eq. (2) is computationally infeasible when the number of families is large (>4). Therefore, we use the following computationally feasible procedure:

1. For each pair of adjacent markers (*l*, *l *+ 1), infer *P*(*α*^(*l*,*l*+1)^, *β*^(*l*,*l*+1)^|**M**^(*l*,*l*+1)^) using the marker data of *all *families. If the number of markers is *L*, this step entails *L *- 1 independent inference problems, independent of the number of families. Thus, the marker data of all families for the two adjacent markers (*l*, *l *+ 1) is used to estimate the parameters *α*^(*l*,*l*+1)^, *β*^(*l*,*l*+1)^. In this step we use set of discrete values for these parameters so that exact computation using the junction tree algorithm [4] is feasible when the number of founders per family is limited. We assume a uniform prior distribution over ***α ***and ***β***.

2. For each family *f*, independently compute the IBD-sharing statistics for marker *l *from

$$P(s_{f}^{l}|M)=\int d\alpha d\beta \sum_{G_{f}}\sum_{s_{NF}\backslash s_{NF}^{l}}P(s_{f}|G_{f})P(G_{f}|M_{f},\alpha ,\beta )\prod_{l}P(\alpha^{\left(l,l+1\right)},\beta^{\left(l,l+1\right)}|M^{\left(l,l+1\right)})$$

where ***s***_*f *_denotes the segregation indicators of the non-founder individuals in family *f*. The integrals are carried out approximately using the set of discrete values for *α *and *β*.

### Application to data

We compared the Bayesian first-order Markov approach, the maximum-likelihood first-order Markov approach, the maximum-likelihood clustered markers approach implemented in MERLIN, and the independent SNPs approach on the 61 families from the Canadian population of GAW15 Problem 2, genotyped with the Illumina marker set. These are affected sib-pair families in which generally only the children have marker data. It has been reported that LD between markers in the 6 K Illumina set appears to be lower than LD between markers in the 10 K Affymetrix set, although significant LD is present [5].

We analyzed chromosomes 1, 2, 3, 4, and 6 in this data set. We estimated the parameters *α*^(*l*,*l*+1) ^and *β*^(*l*,*l*+1) ^for the markers on these chromosomes for which Illumina reports the Decode centimorgan chromosomal location using the procedure described above. For the Bayesian first-order Markov approach, we used a set of ten discrete values for the parameters *α *and *β*, chosen such that they covered 99.9% of the posterior probability mass. They were determined for each pair of adjacent markers independently. The parameters of the maximum-likelihood first-order Markov were determined using the same set of discrete values.

## Results

**Figure 1:**
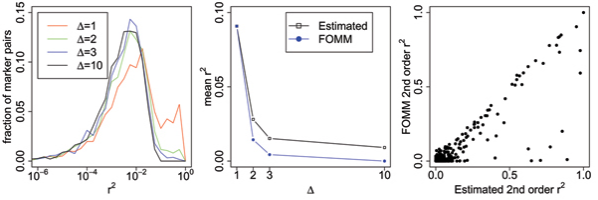
**Assessment of LD between markers**. *r*^2 ^was estimated for adjacent markers, every second marker, every third marker, and every tenth marker (denoted by respectively, Δ = 1, Δ = 2, Δ = 3, and Δ = 10). The left panel shows histogram of *r*^2 ^over marker pairs for different Δ, the middle panel shows mean value of *r*^2 ^as a function of Δ, and the right panel shows scatter plot of *r*^2 ^as modeled by the FOMM vs. *r*^2 ^as estimated from the data for Δ = 2.

To assess the validity of the first-order Markov model (FOMM) for the real data, we estimated LD using *r*^2 ^between adjacent markers, every second marker, every third marker, and every tenth marker (denoted by respectively Δ = 1, Δ = 2, Δ = 3, and Δ = 10). The left panel in Figure 1 shows that most LD is of the order *r*^2 ^~ 10^-2 ^and that strong LD (*r*^2 ^> 10^-1 ^) occurs mostly for Δ = 1 and Δ = 2, and decreases rapidly with Δ. We observe that the low levels of LD with *r*^2 ^~ 10^-2 ^seem to be present independent of Δ. The middle panel shows how well the FOMM captures the LD. The FOMM correctly captures all correlations between adjacent markers (Δ = 1), but has slightly lower *r*^2 ^for Δ > 1. The right panel compares the *r*^2 ^for Δ = 2 as estimated directly from the data with the *r*^2 ^as modeled by the FOMM for Δ = 2, and shows that the FOMM accounts for most of the strong LD between pairs of markers. Thus, the FOMM can handle LD that extends beyond adjacent markers. The FOMM does slightly underestimate low levels of LD (*r*^2 ^< 10^-1^) for Δ = 2, which is a consequence of the fact that correlations in the FOMM scale as r^-Δ^. We conclude that the FOMM is generally valid for the strong levels of LD.

Next, we compare in Table 1 the normalized nonparametric linkage statistic *Z*_*pairs *_for the different approaches. Table 1 shows the difference in *Z*_*pairs *_between any of the approaches and the Bayesian first-order Markov model, which we have taken as the reference (because the data are not simulated there is no gold standard, but we believe that the FOMM assumption is quite accurate). The differences are shown as means over the five chromosomes. The approach that ignored LD yielded the highest scores. There was a difference of 0.242 in the maximum value of *Z*_*pairs *_between the analysis that ignored LD (bottom row) and the Bayesian first-order Markov approach; in four out of five chromosomes the location of the maxima of both approaches differed not more than a few markers. The difference between the Bayesian FOMM approach and the maximum likelihood (ML) FOMM approach was relatively small, in agreement with the fact that the standard deviation in the estimate of *r*^2 ^was only 0.026 ± 0.021, as computed from the Bayesian posterior distribution. As expected, the Bayesian FOMM approach had slightly lower scores than the ML FOMM approach. On average, the Bayesian first-order Markov approach had slightly lower scores than the clustered markers (CM) approach of MERLIN with threshold *r*^2 ^< 0.10, but higher scores than MERLIN with threshold *r*^2 ^< 0.05 to define the clusters. This suggests that the approaches are roughly in agreement, but indicates the problem of specifying the threshold for the clustered markers approach of MERLIN.

**Table 1:** Comparison using all SNPs of chromosomes 1, 2, 3, 4, and 6

		Absolute difference with respect to Bayesian		
			
	Mean difference with respect to Bayesian	Mean	Maximum	Max *Z*_*pairs*_
Bayesian 1^st ^order Markov	NA	NA	NA	1.657
ML 1^st ^order Markov	0.027	0.029	0.115	1.691
Clustered markers *r*^2 ^< 0.10	0.067	0.093	0.914	1.724
Clustered markers *r*^2 ^< 0.05	-0.118	0.231	1.498	1.424
Ind. SNPs *r*^2 ^< 0.10	0.118	0.135	0.4963	1.816
Ignore LD	0.169	0.173	0.659	1.894

**Figure 2:**
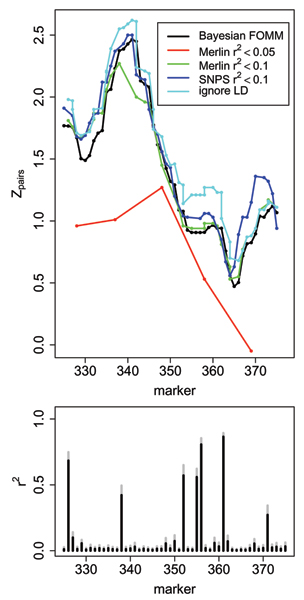
**Comparison of approaches in the region on chromosome 6 with the highest score**. Top panel show nonparametric linkage statistic *Z*_*pairs *_and bottom panel shows estimated pair-wise LD correlation coefficient (*r*^2^) (black) and standard deviation (gray) as estimated from the posterior distribution over the LD parameters computed with the Bayesian FOMM approach.

Figure 2 illustrates this for a region on chromosome 6, where there was a large difference in the scores between MERLIN with threshold *r*^2 ^< 0.10 and MERLIN with threshold *r*^2 ^< 0.05, as well as a decrease in resolution due to the fact that recombination between markers within a cluster is assumed to be zero.

## Discussion

Our data indicate that the FOMM found lower scores than the approach that ignored LD. Further research using simulated data sets is required to assess how these differences are related to false-positive and false-negative rates. An advantage of the first-order Markov model approach is that, in contrast with the other approaches, it does not require the specification of a threshold below which linkage equilibrium is assumed. The FOMM assumes that LD decays exponentially with distance, whereas the clustered markers approach assumes that the LD follows a block structure. Whether the first-order Markov approach or the clustered markers approach is most appropriate will strongly depend on the type and density of the markers, but this can be tested *a priori*. It would be interesting to combine the clustered-markers approach and the first-order Markov model approach, although for larger pedigrees the increased computational effort required may be too high.

As shown, the FOMM can be combined with a Bayesian procedure to allow for uncertainty and/or heterogeneity in the model parameters. However, in the data set considered here we found that this did not have a large effect on the linkage scores. In other populations this might not be the case, for example due to strong population stratification. In the situation in which a smaller number of families are available, it may also be more relevant to take into account the uncertainty.

## Conclusion

We conclude that the application of a first-order Markov model to account for LD between adjacent markers is feasible. For the data set we considered, the assumptions of the FOMM appeared to be generally valid for the strong levels of LD. The effect of accounting for uncertainty and/or heterogeneity in the parameters of the first-order Markov model in a Bayesian approach was small for this data set.

## Competing interests

The author(s) declare that they have no competing interests.

## References

[B1] Kruglyak L, Daly MJ, Reeve-Daly MP, Lander ES (1996). Parametric and non-parametric linkage analysis: a unified multipoint approach. Am J Hum Genet.

[B2] Abecasis GR, Wigginton JE (2005). Handling marker-marker linkage disequilibrium pedigree analysis with clustered markers. Am J Hum Genet.

[B3] Yang X, Huang J, Logue MW, Vieland VJ (2005). The posterior probability of linkage allowing for linkage disequilibrium and a new estimate of disequilibrium between a trait and a marker. Hum Hered.

[B4] Jensen FV (1996). An Introduction to Bayesian Networks.

[B5] Peralta JM, Dyer TD, Warren DM, Blangero J, Almasy L (2005). Linkage disequilibrium across two different single-nucleotide polymorphism genome scans. BMC Genetics.

